# Human emotion recognition with a microcomb-enabled integrated optical neural network

**DOI:** 10.1515/nanoph-2023-0298

**Published:** 2023-10-02

**Authors:** Junwei Cheng, Yanzhao Xie, Yu Liu, Junjie Song, Xinyu Liu, Zhenming He, Wenkai Zhang, Xinjie Han, Hailong Zhou, Ke Zhou, Heng Zhou, Jianji Dong, Xinliang Zhang

**Affiliations:** Wuhan National Laboratory for Optoelectronics, Huazhong University of Science and Technology, Wuhan 430074, China; Optics Valley Laboratory, Wuhan 430074, China; School of Computer of Science and Technology, Huazhong University of Science and Technology, Wuhan 430074, China; Key Lab of Optical Fiber Sensing and Communication Networks, University of Electronic Science and Technology of China, Chengdu 611731, China

**Keywords:** integrated optics, optical computing, optical neural network, optical frequency comb, human emotion recognition

## Abstract

State-of-the-art deep learning models can converse and interact with humans by understanding their emotions, but the exponential increase in model parameters has triggered an unprecedented demand for fast and low-power computing. Here, we propose a microcomb-enabled integrated optical neural network (MIONN) to perform the intelligent task of human emotion recognition at the speed of light and with low power consumption. Large-scale tensor data can be independently encoded in dozens of frequency channels generated by the on-chip microcomb and computed in parallel when flowing through the microring weight bank. To validate the proposed MIONN, we fabricated proof-of-concept chips and a prototype photonic-electronic artificial intelligence (AI) computing engine with a potential throughput up to 51.2 TOPS (tera-operations per second). We developed automatic feedback control procedures to ensure the stability and 8 bits weighting precision of the MIONN. The MIONN has successfully recognized six basic human emotions, and achieved 78.5 % accuracy on the blind test set. The proposed MIONN provides a high-speed and energy-efficient neuromorphic computing hardware for deep learning models with emotional interaction capabilities.

## 1 Introduction

Along with the surge in researches related to artificial intelligence (AI) and massive success in the past decade, deep learning–owing to their ability to mimic human activities such as thinking and decision-making as well as understand human emotion–has become a powerful tool for advanced scientific and industrial applications, including image recognition [1–3], autonomous driving [4–6], and chatbots [7–9]. To achieve smarter applications, the size of parameters in deep learning models is starting to explosively increase, for example, the initial classical models represented by handwritten digit recognition generally have less than a thousand parameters, while the state-of-the-art ChatGPT large language model released by OpenAI already has 175 billion parameters [8], and its next-generation model based on GPT-4 may have up to one trillion parameters. Current deep learning models are typically trained and inferred using digital-clock-based platforms, such as central processing units (CPUs) or graphics processing units (GPUs). They are both highly reconfigurable microelectronic processors that can perform various types of computational tasks, but their clock frequency is only a few GHz [10]. The mismatch between the processor’s computing speed and memory access speed will cause bandwidth bottlenecks that cannot meet the demand for high-speed and low-latency data processing.

Compared with microelectronic devices, photonic devices have the unique advantages of low loss and large bandwidth. Massive amounts of data can be encoded in multiple dimensions of light such as wavelength, amplitude, phase, mode, and polarization by advanced multiplexing techniques to achieve parallel processing [11–21]. Optical neural networks (ONNs) use light as information carrier to implement compute-intense operations in deep neural networks (DNNs), which can significantly improve computing speed and reduce energy consumption. Light propagation can be considered as computation in ONNs. Thus, a specific computational function can be implemented while light propagating through a specially designed dielectric structure or free space. Motivated by these, a myriad of novel hardware structures for optical computing have recently been reported, including coherent architectures based on an on-chip Mach–Zehnder interferometer (MZI) mesh [22–26], incoherent architectures based on wavelength division multiplexing (WDM) techniques [27–33], and diffractive optical frameworks [34–39]. Among these optical computing architectures, the WDM-based scheme has excellent parallelism. Each wavelength can be regarded as an independent channel, and the computational capacity of the system can be expanded by increasing the number of wavelengths. Therefore, this architecture is particularly suitable for tensor computations in practical deep learning models.

However, WDM-based ONNs face significant challenges in complex deep learning applications. Limited by the integration density and computational precision, the ONN-related studies reported so far are mainly demonstrated on simple datasets (e.g., MNIST handwritten digit recognition). The mainstream time-wavelength modulation ONN architectures are bulky because they rely on fiber components, leading to difficulties in integrating them into system-on-a-chip. On the other hand, WDM-based ONN architectures based on on-chip resonant devices (microring [27, 31, 40], [41], [42], nanobeam [43], etc.) are vulnerable to the external environment, and the state of the resonant devices is difficult to maintain accurately, which might introduce additional errors. Moreover, the structure of neural network models is relatively simple, and the problem of error accumulation for optical analog computation in running deep multilayer models has not yet emerged under such model scale.

In this paper, we propose and demonstrate a microcomb-enabled integrated optical neural network (MIONN) architecture with key photonic integrated components, including an integrated optical frequency comb for providing multi-wavelength light source and an on-chip microring weight bank for tensor convolution. The dissipative Kerr solitons (DKS) microcomb generated in a silicon nitride microring cavity provides equally spaced frequency lines with 100 GHz spacing. The microring weight bank consists of a series of configurable microring synapses with self-calibrating capability. Thanks to the feedback control, the weighting precision of microrings can achieve 8 bits. To evaluate the proposed MIONN, we perform two proof-of-concept experiments, including parallel image convolution and human emotion recognition. Large-scale pixels of images can potentially be processed at the speed of light with the throughput of 51.2 TOPS and high energy efficiency of 4.18 TOPS/W. The results of image convolution are comparable to that of a 64-bit digital computer, and the human emotion recognition task has achieved 78.5 % accuracy on the blind test set. Six basic human emotions can be successfully recognized in single image test. Our MIONN provides a scalable neuromorphic architecture to implement tensor computations in practical deep learning models, and presents a promising avenue towards deploying photonic-electronic AI hardware in modern scientific and industrial applications.

## 2 Principle

**Figure 1:**
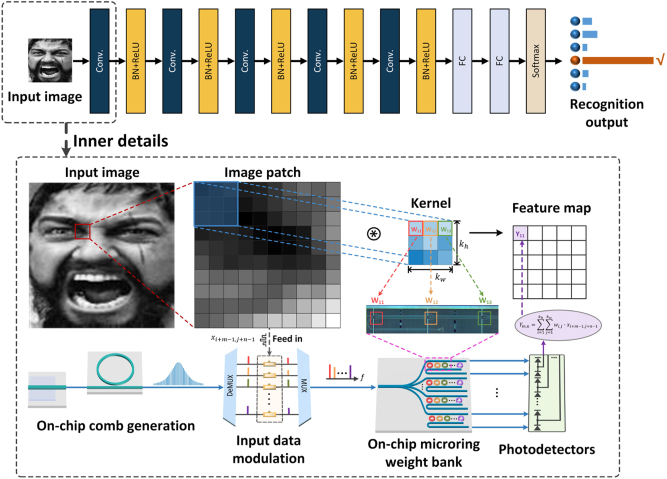
Conceptual drawing of the MIONN for human emotion recognition. Convolution operations in CNN models for human emotion recognition can be implemented at light speed in the MIONN. The DKS microcomb containing a series of equally spaced frequency lines is generated in the silicon nitride microring cavity as a multi-channel light source for the MIONN. The data of input images is encoded on the intensity of each frequency line through electro-optic modulation as the input of the on-chip microring weight bank. The weight parameters of the convolutional kernels are configured on the microrings of the on-chip weight bank, and the weight values can be changed on demand by adjusting the state of microrings. Photodetectors receive the output of on-chip microring weight bank and perform weighted summation operation. DeMUX: wavelength division demultiplexer, MUX: wavelength multiplexer.

Physically implementing compute-intense operations in neural network models is a novel computational paradigm. Our proposed MIONN architecture uses light as an information carrier and can perform tensor convolution operations. Figure 1 shows the basic principle of the MIONN and a convolutional neural network (CNN) model of human emotion recognition. This CNN model consists of convolutional layers, nonlinear layers (including batch normalization and ReLU nonlinear activation), and fully-connected layers. A DKS microcomb generated in a silicon nitride microring serves as the multi-channel light source, and these equally spaced frequency lines are shaped in intensity using a WaveShaper, and then divided by a wavelength division demultiplexer to independently carry normalized data from input images. An external arbitrary waveform generator is used to encode the image information in the electro-optic (EO) modulators. Normalized grayscale values from input images are intensity-encoded in the waveform in radio frequency (RF) domain via an arbitrary waveform generator, and then amplified by an electronic driver. After that, the input data encoded in the RF waveform is fed in the intensity modulator array with different frequency lines. Discrete and modulated frequency lines can be regarded as a series of independent signal channels, and they are multiplexed in the bus fiber and then coupled into the on-chip microring weight bank.

The weight parameters of convolutional kernels are configured on the on-chip microring weight bank by applying programmable voltages to the microheaters of each microring. The on-chip microring weight bank is composed of *N* rows of microring synapses that correspond to *N* convolutional kernels to implement tensor convolution, and the size of synapses can be flexibly scaled up or down to match different kernel size. Since microrings are sensitive to thermal variations and inter-channel cross talk, we specially developed a self-calibration approach to maintain the weight stability. The convolution computation is completed when the modulated light propagates through the on-chip microring weight bank, and the weighted summation operation is achieved in photodetectors (PDs). Because the on-chip microring weight bank is configured with multiple different convolutional kernels at one time, multiple different feature maps can be obtained by only one optical tensor convolution operation.

**Figure 2:**
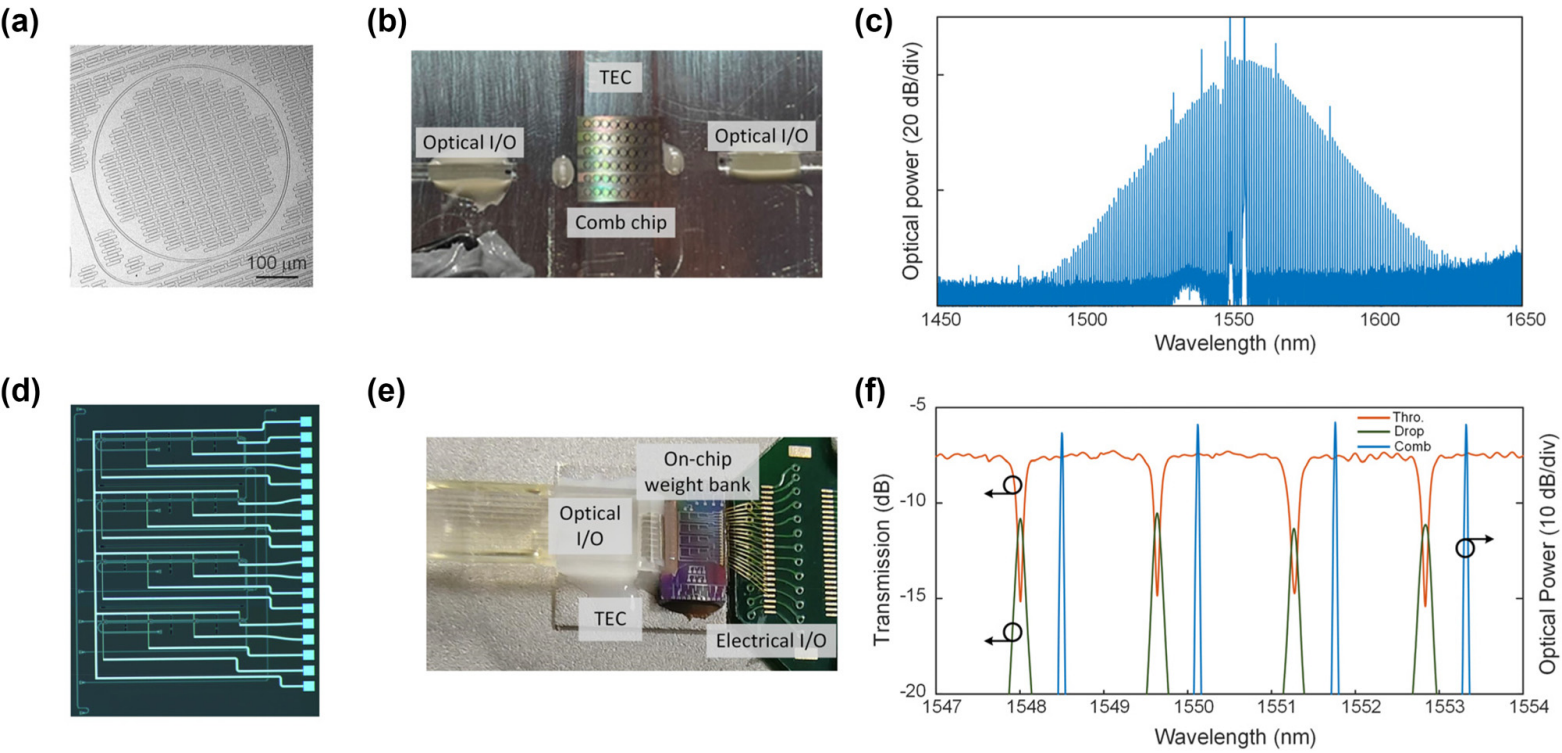
Design of the DKS microcomb and the on-chip weight bank in MIONN. (a) Microscope image of the silicon nitride microring cavity. (b) Photograph of the packaged comb chip. (c) Measured optical spectrum of microcomb. Four frequency lines near 1550 nm are selected for the proof-of-concept experiments. (d) Microscope image of the on-chip microring weight bank. (e) Photograph of the packaged microring weight bank chip. (f) The transmission spectra of the microring synapse at the through and drop ports and the optical power of the shaped frequency lines in the optical spectral snapshot.

Benefiting from the high refractive index, high nonlinear coefficient, and high damage threshold, the silicon nitride micro-cavity is suitable for generating nonlinear optical phenomena at high power laser input. Figure 2(a) shows a microring cavity fabricated on the silicon nitride platform, which is available at commercial photonic foundries. The silicon nitride microring cavity used for on-chip comb generation has a width-height cross section size of 1.65 × 0.8 μm^2^ and a free spectral range (FSR) of 100 GHz. The auxiliary laser heating method [44] is adopted to quickly generate and precisely tune the DKS microcomb, and an automatic feedback algorithm is developed to compensate for external variations such as frequency noise, frequency drift and power fluctuations to ensure that the comb state could be maintained for up to several hours. Figure 2(b) shows the photograph of the packaged silicon nitride microring cavity chip with optical input/output (I/O) and the thermoelectric cooler (TEC). Polarization-maintaining fiber is used in optical I/O for stable external driving [45], and the TEC is mounted below the chip to precisely control the temperature. The optical spectral snapshot of the generated Kerr frequency comb with the single DKS state is depicted in Figure 2(c), and four frequency lines near 1550 nm are selected for the proof-of-concept experiments.

Different from electronic processors, our MIONN performs convolution operations on the integrated photonics platform. The 2D convolution operation (stride = 1) of the input image *X* and *k*_*h*_ × *k*_*w*_ convolutional kernel *W* can be mathematically expressed as

(1) $$\begin{array}{c} Y_{m,n}=\overset{k_{h}}{\underset{i=1}{\sum }}\overset{k_{w}}{\underset{j=1}{\sum }}w_{i,j}\cdot x_{i+m-1,j+n-1} \end{array}$$

where (*m*, *n*) is the position coordinate value of the convolutional result *Y*_*m*,*n*_ in the output feature map, *w*_*i*,*j*_ is the weight parameter of the convolutional kernel, *k*_*h*_ is the height of the convolutional kernel, and *k*_*w*_ is the width of the convolutional kernel. To visualize how the original image is encoded in different wavelength channels and the mapping of the convolutional kernel and the microring weight bank, we provide a schematic of the working data flow in Supplementary Note 1.

Figure 2(d) shows the micrograph of the microring weight bank chip, which is fabricated by a commercial photonic foundry with a standard silicon-on-insulator (SOI) process. Details of device fabrication can be found in Supplementary Note 2. The microring weight bank is comprised of *N* microring synapses, and each synapse corresponds an individual *k*_*h*_ × *k*_*w*_ convolutional kernel, thus the equivalent dimensions of this microring weight bank are [*N* = 4, *k*_*h*_ = 2, *k*_*w*_ = 2]. The height and width of convolutional kernel can be set as desired, as long as the product of *k*_*h*_ × *k*_*w*_ does not exceed the number of microrings in a synapse. Importantly, the equivalent dimensions [*N*, *k*_*h*_, *k*_*w*_] can also be flexibly extended by increasing the number and size of microring synapses in the weight bank to enable massive tensor convolution. Thermo-optic phase shifters made of TiN heaters are used to tune the microrings, and the weight parameters of each microring can be precisely configured by setting corresponding voltages on its heater. Thermal isolation trenches are employed between each microring of the weight bank to reduce the thermal cross talk. The packaged weight bank chip (see in Figure 2(e)) has optical and electronic I/O for light propagation and weight parameter control. Optical signals that encoded the image data enter and leave the weight bank chip via vertical grating couplers, and programmed voltage control is applied to the on-chip heaters through wire bonding. The TEC module is mounted below the weight bank chip to compensate temperature variations. Figure 2(f) displays the transmission spectra of the microring synapse at the through and the drop ports and the optical power of the shaped frequency lines. Detailed characterization of the microring in the on-chip weight bank can be found in Supplementary Note 3.

## 3 Results

In this section, we first experimentally validate the effectiveness of high-precision photonic tensor convolution using the proposed MIONN, including the systematic feedback control and the tensor convolution results. Next, we demonstrate the successful recognition of six human emotions using the MIONN.

### 3.1 High-precision photonic tensor convolution using MIONN

Precision is an important metric to evaluate the analog computing hardware, especially when facing large human-computer emotional interaction models and complex computing tasks. As an analog computing architecture, the optical comb generation and the on-chip weight bank of the MIONN are both realized by microring cavity chips. Because microring cavity is a compact resonant device, its state is sensitive to fabrication and environmental variations, including fabrication errors, frequency drift, optical power fluctuations, thermal crosstalk, etc. Therefore, MIONN requires customized automatic feedback control to ensure high computational precision. Here, we specifically designed a systematic feedback control procedure for MIONN to adjust the photonic devices of each functional module in real time by monitoring related optical parameters in the whole optical link. There are two procedures to maintain the system stable. One is to maintain the stability of the generated DKS microcomb, and the other is to calibrate the weight of each microring in the on-chip weight bank.

**Figure 3:**
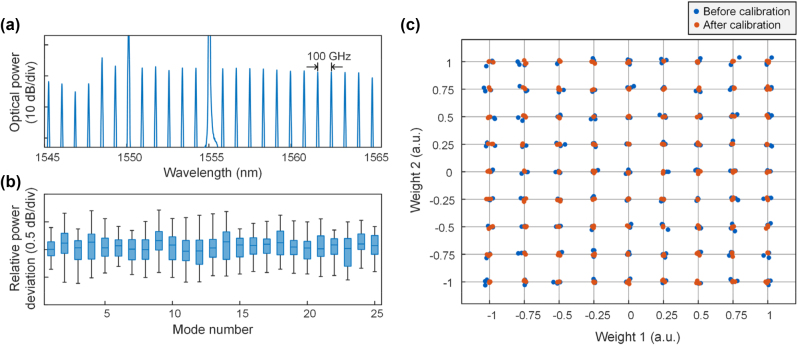
Systematic feedback control for the MIONN. (a) Optical spectra of the DKS microcomb at single soliton state with a FSR of 100 GHz. (b) Power deviations of the 25 central frequency lines of the microcomb. (c) Comparation of the weighting precision in a two-microring synapse before and after calibration. Each blue/red dot represents a measured weight before/after calibration, and each point on the grid is tested three times.

For the DKS microcomb, the auxiliary laser heating method [44] is adopted to quickly generate the microcomb. Figure 3(a) shows 25 central frequency lines of the DKS microcomb at single soliton state. To maintain the single soliton state for a long time, our feedback procedure uses the total power of the microcomb to compensate for the effect of environmental variations. Particularly, the total power of the single soliton state is regarded as the threshold value, and we define a figure of metric (FOM) as the difference between the real-time power of the microcomb and the threshold value. When the FOM is less than zero, the feedback control is activated and all resonances are thermally red-shifted while the auxiliary laser is tuned closer to the resonance peak, then the pump laser can re-enter the cavity from the red detuning region and trigger the DKS burst. The power deviations of the 25 central frequency lines of the microcomb in Figure 3(a) within 60 min are measured, and the results of statistical analysis are depicted in Figure 3(b). Results show that the power fluctuation of the central frequency lines are all less than 2 dB, and most relative power deviations are less than 1 dB, which proves the validity of the feedback control and ensures high computational precision of the MIONN.

Efficient calibration of the on-chip weight bank is the other key to ensure the precision of the MIONN. For every microring in the on-chip weight bank, a one-to-one mapping between the configured weights and the applied voltages is established before the calibration. We specially developed a self-calibration procedure that works in concert with the circuit hardware to monitor and calibrate the microring weight bank (see the Supplementary Note 4 for details). To achieve high-precision voltage scanning, customed power supply (see the Supplementary Note 5 for details) with 16-bit resolution to ensure accurate voltages applied to the microring, and the technical information of the balanced photodetector is given in the Supplementary Note 6. The weight of each microring can be precisely adjusted and corrected once the current measured weight does not match the set weight. Figure 3(c) shows the test result on a mesh-plot. This mesh-estimation is a common method to visually describe the weighting precision of microrings [40, 42, 46], in which two microrings are configured to equidistant grid points of (*w*_1_, *w*_2_), *w*_1,2_ ∈ [−1, 1], and the weight precision is evaluated at each grid point. Grid points correspond to combinations of weight, and each combination is tested three times. From the test results, we observe that the calibrated weights are closer to the set weights, and the weighting precision can reach 8 bits (the estimation method can be found in Ref. [42]), verifying the validity of the self-calibration procedure.

**Figure 4:**
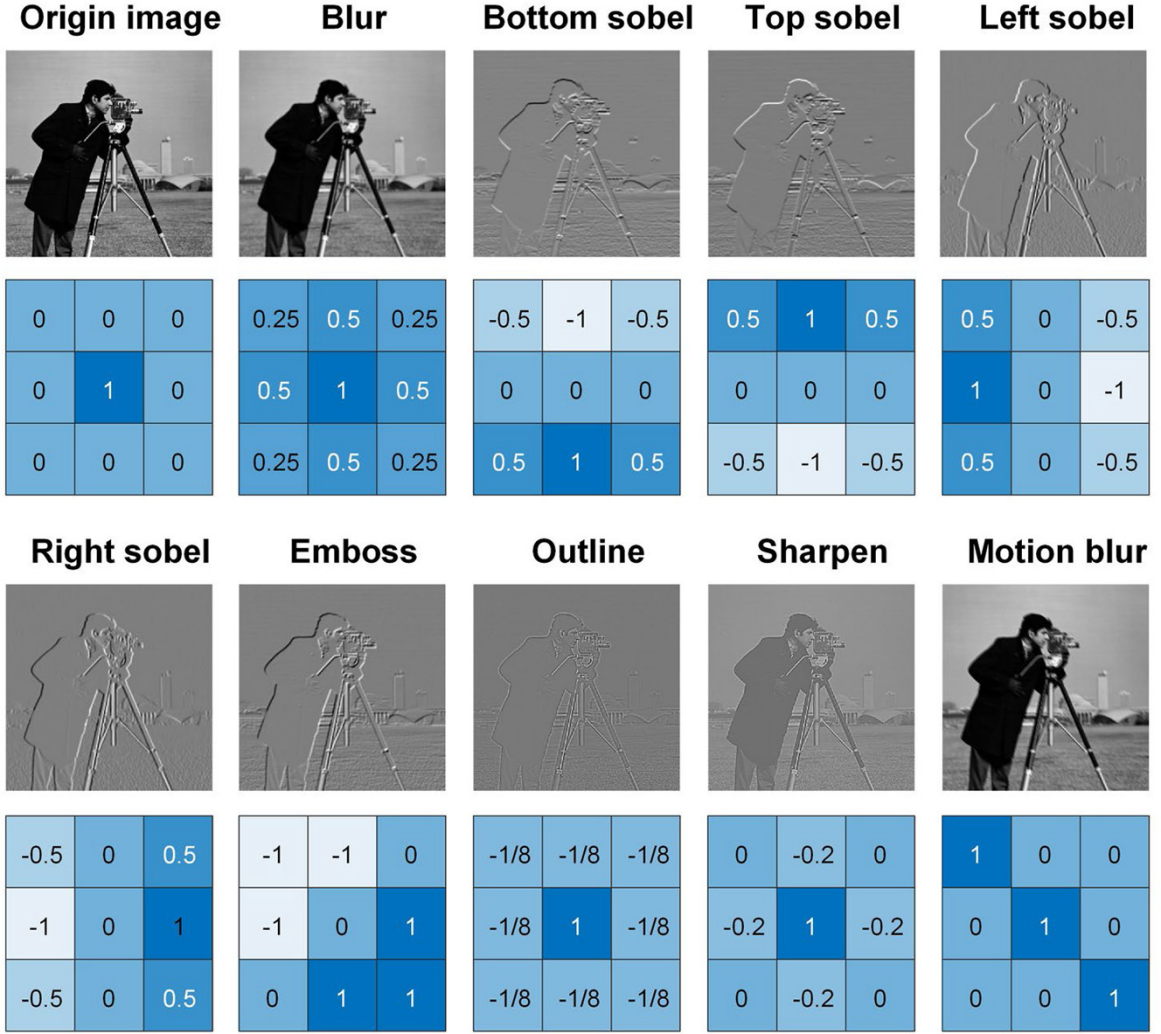
Tensor convolution results implemented by the MIONN. The standard test image is convolved with nine different kernels, including classical image processing (blur, motion blur, emboss, outline, sharpen) and edge extraction with bottom/top/left/right Sobel operator.

Image convolution is a fundamental operation in CNN models, which is used to learn spatial patterns and features from input images. The image convolution operation can be expressed as the weighted summation of element-wise products of a small matrix (called the convolutional kernel) with corresponding image patch in the input image, i.e., the kernel weights are assigned to pixel values of the input image. In each image convolution operation, the convolutional kernel slides over the input image with preset step length and the weighted summation results are reshaped to a feature map. By decomposing the original matrix into the positive matrix and the negative matrix [30], the computational domain can be extended from the domain of positive numbers to the domain of real numbers. Details on the photonic matrix multiplication including negative number are given in Supplementary Note 7. To validate the convolution performance of the MIONN, we use a 256 × 256 grayscale ‘Cameraman’ image with 8-bit color depths as the standard test image, and nine commonly used convolutional kernels are employed to extract its different features. In the MIONN, each frequency line of the microcomb represents a signal channel used for loading multi-channel input images. The grayscale values of the pixels of input images are normalized and then arranged as the serial data flow fed in 10 GHz-bandwidth electro-optic modulators. After temporal modulation, these pixel values are encoded in the intensities of frequency lines and then weighted by the on-chip microring weight bank during light propagation. Optically-weighted results are received by the high-speed PDs, in which the power of overlapped frequency lines is added as the optical convolution results and then reshaped to final feature maps. Figure 4 presents the optical convolution results of the standard test image convolved with nine different kernels, including classical image processing and edge extraction. The Sobel operator is used as the kernels for image edge extraction to compute the gradient approximation of the image intensity at each pixel. We observe that the feature maps yielded by optical convolution successfully implement different image processing (blur, motion blur, emboss, outline, sharpen) and highlight the sharp edges in the origin image in bottom/top/left/right direction. Experimental results closely resemble the theoretical feature maps generated by a 64-bit digital computer, which verifies the capability of the MIONN to perform tensor convolution.

### 3.2 MIONN for human emotion recognition

**Figure 5:**
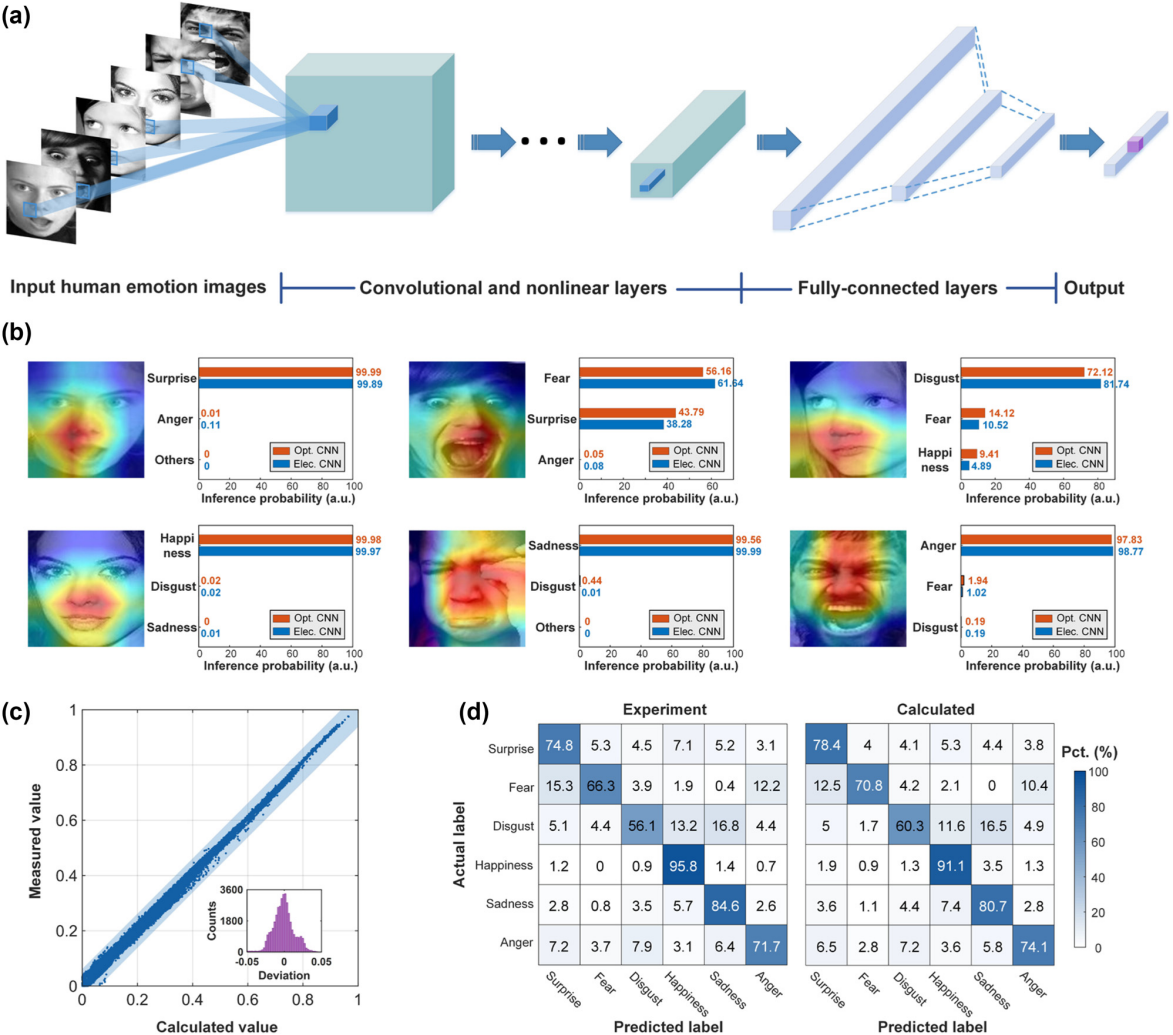
Human emotion recognition of the RAF-DB dataset using the MIONN. (a) The processes and structure of the CNN model for human emotion recognition. (b) The class activation mappings (CAMs) and the top-3 probability of human emotion images. The MIONN and a 64-bit digital computer are used for the recognition of six basic human emotions, including surprise, fear, disgust, happiness, sadness, and anger. The red bars in the figure represent the probabilities obtained by optical inference, and the blue bars represent the probabilities obtained by electrical inference. (c) Scatter plot for convolution accuracy measurement with a fixed kernel matrix. The inset is a deviation distribution histogram showing the difference of the measured values and the calculated values. (d) The confusion matrices of human emotion classification of the RAF-DB dataset. The accuracy of the prediction results for experiment and calculation shows good agreement.

Beyond the multi-channel image convolution, we further demonstrate a CNN model to recognize human emotion in the real-world affective faces (RAF-DB) database [47]. Importantly, our MIONN is fully reconfigurable and scalable, thus in addition to convolutional layers, average pooling and fully connected layers can also be implemented. The matrices involved in pooling and convolution layers are usually large and difficult to be computed at once on the optical hardware, so hardware-friendly algorithms are needed to decompose the large matrices into smaller ones. A matrix decomposition algorithm dedicated to microring weight bank is proposed in our previous work [30] and can be used in optical CNN models. Since the goal of our task is to recognize human facial expressions with rich details, versus handwritten digit images or two-digit recognition, both the size of images in database and the complexity of the CNN model in our experiments are challenging. Figure 5(a) shows the processes and structure of the CNN model used in our experiment, including five convolutional layers, five nonlinear layers (including batch normalization and ReLU nonlinear activation), and two fully-connected layers. The nonlinear activation function in this work is performed by the electronic hardware. We randomly choose 12,000 human emotion images from the RAF-DB dataset and split them into the training set (10,000 images) and testing set (2000 images). The input image is a matrix with a size of 100 × 100, which is much larger than handwritten digit images in the MNIST dataset. Details on the CNN training can be found in Supplementary Note 8. The parameters of CNN are firstly trained with a standard back-propagation algorithm on a digital computer and the MIONN is used to implement convolutional layers and fully-connected layers in the inference phase of single human emotion image.

In the MIONN, the 100 × 100 pixels, 8-bit grayscale images of human emotion are first converted to optical data flow using optical intensity modulation and then fed into the convolutional layer implemented by on-chip microring weight bank. Each four-microring photonic synapse in the on-chip weight bank represents a kernel in the convolutional layer, and four photonic synapses can perform convolution operation simultaneously. The convolution layer first convolutes the input images with four photonic kernels and the results are then processed with batch normalization and ReLU nonlinear activation to generate feature maps. As the kernels slides along the input images, the photonic synapses can extract key features used for recognition. To visualize which facial features the photonic synapses are sensitive to, class activation mappings (CAMs) of six different human emotion images are yielded and depicted in Figure 5(b). These CAMs display the sensitive areas of photonic synapses by heat map, which helps us investigate key features that contribute the recognition results. After serializing into a feature vector through flatten operation, the convolutional results are fed into the fully-connected layer and then the final output is obtained by the softmax function. The final output gives the predicted probabilities of the six emotion labels of the input image, and the largest of them is taken as the recognition result. Figure 5(b) also provides the top-3 probability of six human emotion images obtained by both the MIONN and a typical 64-bit digital computer (Intel(R) Core(TM) i9-12900K CPU, 64 GB RAM). By extracting key features of the human face such as mouth, nose, and the corner of the eyes using photonic synapses, the MIONN has successfully achieved the recognition of six basic human emotions and the recognition results are comparable to those of digital computers.

To further evaluate the computational accuracy of the MIONN, the human emotion image labelled as ‘anger’ is chosen to execute convolution operation in the first convolutional layer, and the comparison of the optical and theoretical convolution results are presented in Figure 5(c). The scatter points are closely distributed along the diagonal line and most of the points are within the blue shaded area, i.e., the deviations are within the range of [−0.05, 0.05], indicating that the measured and theoretical values are in excellent agreement. We then randomly feed 1000 images from the RAF-DB human emotion dataset into both the MIONN and the digital computer to test the accuracy classification tasks. Since the size of the input matrix is much larger than the MNIST handwritten digital dataset, which is frequently used to benchmark in the field of optical computing (see Supplementary Note 9 for details), we only perform the first convolutional layer with the microring weight bank for proof-of-concept in this test. The diffusion matrices with six categories of human emotion (‘surprise’, ‘fear’, ‘disgust’, ‘happiness’, ‘sadness’, and ‘anger’) obtained by both the MIONN (marked as ‘experiment’) and the digital computer (marked as ‘calculated’) are displayed in Figure 5(d). The blind test set accuracies of the MIONN and the digital computer are 78.5 and 82.3 %, respectively. We simulate the impact of Gaussian noise with different standard deviations on the inference accuracy of CNN and find that only when the standard deviation of Gaussian noise is less than 0.1, the inference accuracy does not decrease significantly (see Supplementary Note 10 for details). Results show that the classification accuracy of the MIONN is comparable to that of a digital computer, and the ability of the MIONN to recognize human emotions is verified.

## 4 Discussion

In our proof-of-concept experiments, the MIONN demonstrate the successful recognition of human facial expression, which is one of the most effective and universal ways for humans to express emotions and intentions. To take full advantage of the parallelism of photonic computing, we use an integrated DKS microcomb as the light source, and tens of generated frequency lines can be used as independent channels to load data to support optical tensor convolution of large size input images with richer details. On-chip microring weight bank with compact footprint can be straightforwardly configured to simulate the weights of convolutional kernels. Thanks to calibration procedures specifically designed for optical frequency comb and microring weight bank, our MIONN achieves a weighting precision of up to 8 bits, which is comparable to state-of-the-art electronic neuromorphic processors. In addition, the MIONN architecture is highly scalable, the number of channels and the size of the on-chip weight bank can be flexibly scaled, and its throughput can be significantly improved by adopting frequency lines with smaller intervals and more compact microring layout.

**Figure 6:**
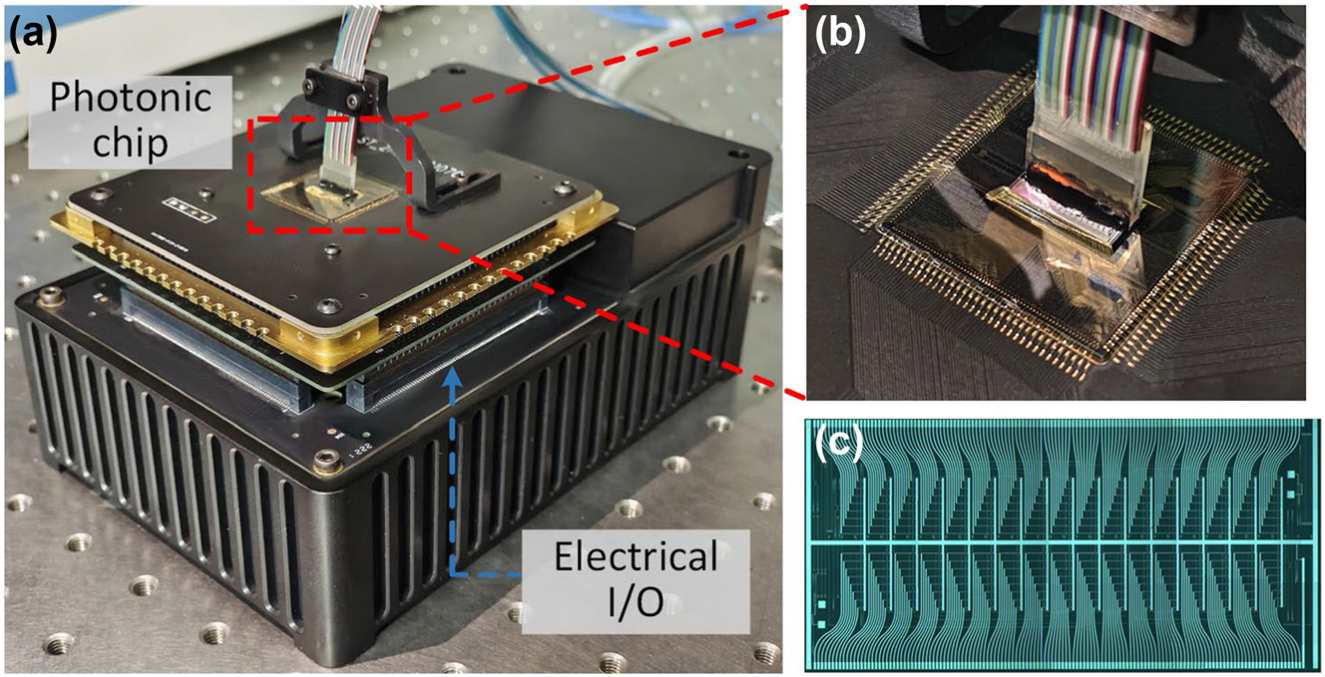
Prototype of photonic-electronic AI computing engine. (a) The photonic-electronic AI computing engine packaged with complete optical and electrical I/O. 512-channel high-speed electrical I/O is achieved through standard pluggable connecting finger interconnected with external control circuits instead of bulky Jumper wires. (b) The zoom-in photo of the packaged photonic chip. (c) Micrograph of the 16 × 16 on-chip microring weight bank.

To demonstrate the scalability of the MIONN architecture, we further develop a packaged 16 × 16 photonic weight bank chip and a base with integrated circuit control modules. In this prototype, the photonic chip and electrical base can be considered as a photonic-electronic AI computing engine for high-throughput tasks. Figure 6(a) shows the photonic-electronic AI computing engine packaged with complete optical and electrical I/O, and the zoom-in photo of the photonic chip is shown in Figure 6(b). Optical signals are connected on and off the chip through a vertically coupled grating array, and 512-channel high-speed electrical I/O is achieved through standard pluggable connecting finger interconnected with external control circuits instead of bulky Jumper wires. Figure 6(c) displays the micrograph of the photonic chip, each microring in the 16 × 16 on-chip weight bank has an equal spacing of 0.14 mm, so the footprint of a microring is 0.14 × 0.14 = 0.0196 mm^2^. The structure of the microring is designed to match the frequency lines with 50 GHz interval, providing more than 80 channels available in the commonly used C-band to support AI applications requiring high throughput. Since the microcomb chip with a frequency spacing of 50 GHz is to be fabricated, systematic test of the photonic-electronic AI computing engine has not been conducted for the time being.

Table 1 summarizes the comparison between our MIONN architecture and other neuromorphic computing hardware in terms of key performance metrics. Due to the scalability, we evaluate the MIONN with small-scale (*N* = 4) and large-scale (*N* = 16) on-chip microring weight bank. The small-scale MIONN can achieve a throughput of 3.2 TOPS, a computing density of 10.2 TOPS/mm^2^, and an energy efficiency of 1.21 TOPS/W. As the scale increases, the advantages of throughput and energy efficiency of MIONN architecture begin to emerge. Large-scale MIONN has the potential to support a throughput of 51.2 TOPS and a high energy efficiency of 4.18 TOPS/W. The energy consumption here does not include external benchtop instruments such as arbitrary waveform generator, oscilloscope, and the computer used to train neural networks. We also conduct statistics on the overall energy consumption with external benchtop instruments, and detailed information on key metrics of the MIONN can be found in Supplementary Note 11. Since the throughput is directly proportional to the size of the on-chip weight bank, the scalable MIONN architecture can be designed with highly flexible scaling to meet the needs of different application scenarios. The reconfigurable and compact microring is an ideal basic unit for neuromorphic computing because it can scale up largely, and thus support high computing density architecture. Since the process of optical computing is almost completely passive and only requires very low power consumption to maintain the resonant state of microrings, the MIONN has high energy efficiency. In contrast to bulky space diffractive systems and fiber optic systems, the key components of MIONN are integrated and can be fabricated in CMOS-compatible platforms, which is important for the miniaturization and commercialization of computing hardware.

**Table 1:** Comparison of the MIONN and other neuromorphic computing hardware.

Technology	Throughout (TOPS)^a^	Computing density (TOPS/mm^2^)^b^	Energy efficiency (TOPS/W)^b^	Precision (bits)	Integration
MZI mesh [22]	6.4	N/A	N/A	8	Yes
MZI mesh [26]	7.2	9.88	1.84	4	Yes
MRR array [48]	1.6	4.21	0.7	>5.5	Yes
MRR array [31]	2.4	13.88	0.3	7	Yes
WDM + PCM [28]	28.8	1.2	0.4	5	Yes
InP SOA [49]	1.6	N/A	0.59	4.5	Yes
WDM + ONN [29]	11.32	N/A	1.25	8	No
This work	3.2 (*N* = 4)	10.2	1.21 (*N* = 4)	8	Yes
	51.2 (*N* = 16)		4.18 (*N* = 16)		

^a^The throughput is estimated at a typical detection rate of 100 GHz. ^b^Regarding only the optical comb and weight bank chips.

Furthermore, the key performance metrics of MIONN architecture can be further improved, and here we discuss several feasible ways to further exploit the potential of MIONN. Implementing nonlinear activation functions in the optical domain can avoid frequent photoelectric conversions and improve the practicability of optical neuromorphic hardware. Although the nonlinear activation function in this work is performed by the electronic hardware, two type of optical nonlinear function chips based on Ge/Si hybrid structure have been successfully experimentally demonstrated in our recent works [50, 51], and will be integrated into the MIONN architecture as an important module to compensate for the lack of optical nonlinear functions. To reduce the power consumption, the on-chip comb generation can reach as low as 98 mW [52] using extremely low loss silicon nitride waveguides. On the other hand, conventional thermo-optical phase shifters can be replaced by phase shifters based on phase change material (PCM). PCM-based devices can operate without static energy consumption [28, 53, 54], and PCM only requires energy when switching to different states. High-speed germanium-on-silicon PDs can be directly integrated to the on-chip weight bank to reduce computing latency [55]. In addition, external control circuits can be connected to the photonic chip through advanced 3D stacking or optoelectronic fusion technologies to achieve higher I/O rate and higher integration.

## 5 Conclusions

In summary, we propose a photonic-electronic MIONN architecture based on CMOS-compatible platform for neuromorphic computing. The MIONN computes through light propagation, and naturally has higher parallelism and lower energy consumption compared to traditional electrical neural networks. Enabled by dozens of channels provided by the integrated optical microcomb, we develop a prototype photonic-electronic AI computing engine with a potential throughput up to 51.2 TOPS, a computing density of 10.2 TOPS/mm^2^, and a high energy efficiency of 4.18 TOPS/W. The auxiliary laser heating method and automatic feedback control ensure rapid generation and long-term stability of the microcomb (less than 2 dB power fluctuation within 60 min), and a self-calibration procedure for the on-chip microring weight bank is developed to achieve 8 bits weighting precision comparable to that of microelectronic neuromorphic processors. In proof-of-concept experiments, our MIONN is able to perform tensor convolution operations with high accuracy in the optical domain, and can run a multi-layer CNN model to successfully recognize human emotion images with a blind test set accuracy of 78.5 %. The proposed MIONN provides a promising direction for next-generation computing hardware for compute-intense AI applications.

## Supplementary Material

Supplementary Material Details
