# Supplementary material for: Human emotion recognition with a microcomb-enabled integrated optical neural network
Source: Nanophotonics. 2023 Oct 2;12(20):3883–94. doi: 10.1515/nanoph-2023-0298 (PMC11501890; doi:10.1515/nanoph-2023-0298)
Supplement: Supplementary file 1 — Supplementary Material Details [file j_nanoph-2023-0298_suppl_001.pdf]

Supplementary Materials for

Human emotion recognition with a microcomb-enabled integrated optical neural network

Junwei Cheng[1,2†], Yanzhao Xie[1†], Yu Liu[3†], Junjie Song[1], Xinyu Liu[1], Zhenming He[1], Wenkai Zhang[1], Xinjie Han[4], Hailong Zhou[1], Ke Zhou[1], Heng Zhou[4], Jianji Dong[1,2,*] and Xinliang Zhang[1,2]

[1]Wuhan National Laboratory for Optoelectronics, Huazhong University of Science and Technology, Wuhan 430074, China

[2]Optics Valley Laboratory, Wuhan 430074, China

[3]School of Computer of Science and Technology, Huazhong University of Science and Technology, Wuhan 430074, China

[4]Key Lab of Optical Fiber Sensing and Communication Networks, University of Electronic Science and Technology of China, Chengdu 611731, China

[†]*These authors contributed equally to this work.*

**Correspondence E-mail: jjdong@mail.hust.edu.cn*

Contents

S1. Schematic of the working data flow 2
S2. Device fabrication 2
S3. Characterization of the microring and the modulator 3
S4. Control flow of the self-calibrating microring 4
S5. Design of multi-channel power supply 5
S6. Technical information of the balanced photodetector 7
S7. Photonic matrix multiplication including negative values 7
S8. Details of CNN training 8
S9. Comparison of datasets used for photonic experimental validation 9
S10. Impact of Gaussian noise on CNN inference accuracy 9
S11. Estimation of throughput, computing density, and energy consumption 10
References 11

S1. Schematic of the working data flow

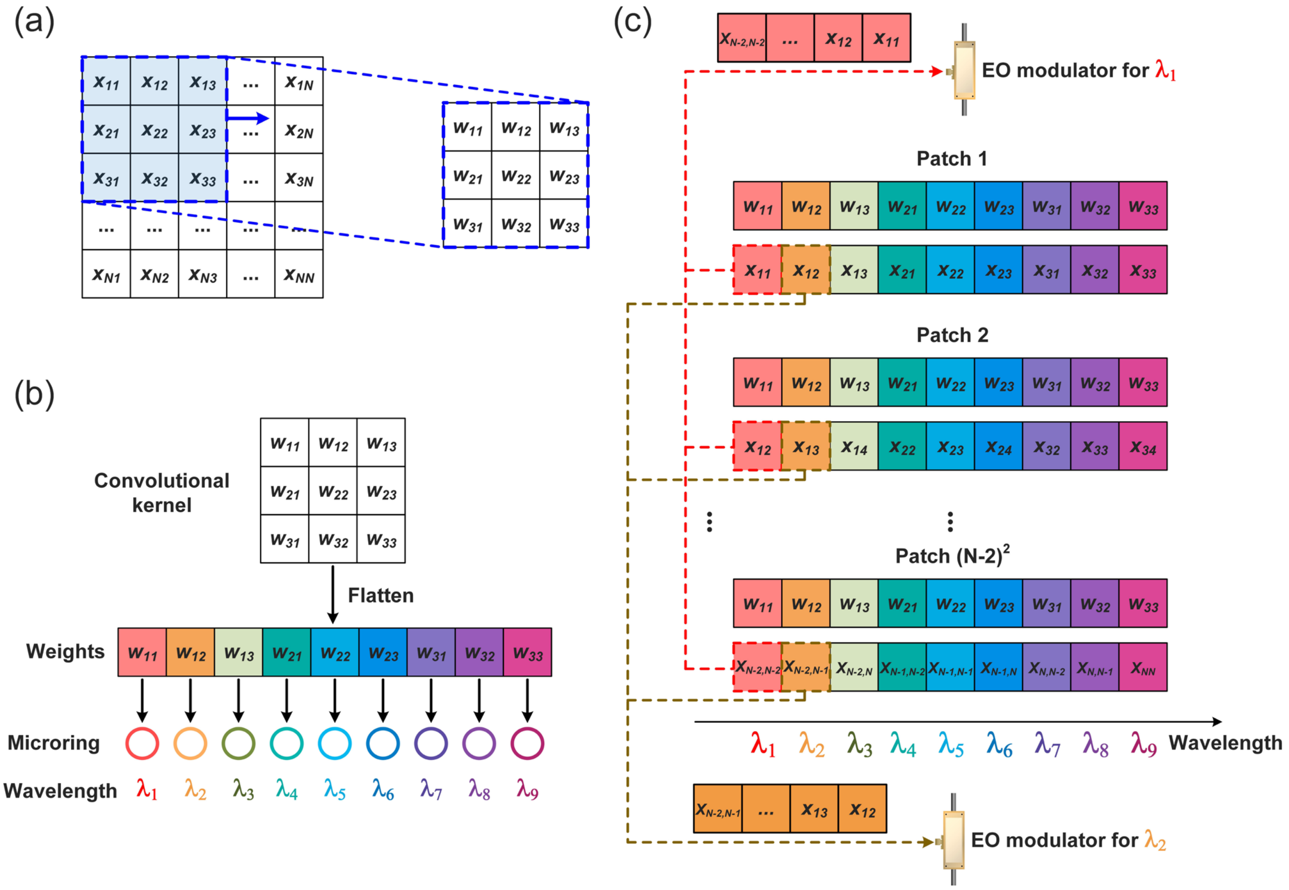

Fig. S1. Schematic of the working data flow (stride=1). **a** 2D convolution operation of a N×N input image and a 3×3 convolutional kernel. **b** Mapping between the convolutional weight parameters and the microrings/wavelengths. **c** How the original image is encoded in different wavelength channels and fed in the corresponding EO modulator.

S2. Device fabrication

The DKS microcomb is generated by optical parametric oscillation in a single microring cavity, which is fabricated on a CMOS-compatible silicon nitride platform. Low-pressure chemical vapor deposition (LPCVD) was used to deposit silicon nitride thin film. The designed device pattern is transferred onto photoresist by electron beam lithography (EBL) and developing, and then transferred to silicon nitride thin film by induction plasma etching (ICP). The fabricated silicon nitride micro-ring cavity has a waveguide cross section of 1.65 μm × 0.8 μm (width × height), a Q-factor of 1 million, and a free spectral range of 100 GHz.

On-chip microring weight bank is fabricated on an SOI wafer with a 220 nm thick top silicon layer and a 3 μm thick buried oxide (BOX). The designed layout is transferred onto photoresist using EBL and the top silicon is etched by ICP. The grating coupler is shallowly etched by 70 nm, while the silicon waveguide is fully etched by 220 nm. Between the waveguide and metal electrodes, 1 μm thick

silicon dioxide was deposited using plasma enhanced chemical vapor deposition (PECVD). The metal for the heaters and routing is deposited by electron beam evaporator (EBE). A thin layer of TiN is deposited as a resistive layer for the heaters, and a metal film of AlCu is patterned as the electrical connection to the electrodes and heaters. Heat insulation trenches are etched between adjacent microrings to reduce thermal cross talk.

S3. Characterization of the microring and the modulator

Fig. S2(a) shows the microscope image of an add-drop microring in the on-chip weight bank, which is fabricated with a standard SOI process. The heater is patterned on the microring and the resonant wavelength of the microring can be controlled by changing the voltage applied to the microring. To verify the tunability of the microring in a four-ring synapse, we first apply a base voltage to four microrings to shift their resonant wavelength to the initial operating position, and then gradually increase the voltage applied to the second microring. The transmission spectrum of the drop port of the four-ring synapse is shown in Fig. S2(b). We can observe that only the second resonant peak moves, while the other three peaks remain unchanged, indicating that the inter-ring thermal cross talk is well suppressed. Before starting the computation task, the microring and the modulator need to be calibrated separately. Fig. S2(c) illustrates the power variation of the through and drop ports of the microring as the applied voltage increasing. After the differentiation and normalization of the two, the mapping of the microring weight and voltage can be obtained, as shown in Fig. S2(d). Since the difference operation produces negative numbers, the weights of the microring are in the range [-1, 1]. Fig. S2(e) shows the mapping of the weight of the modulator to the voltage. Since the light intensity cannot be negative, the modulator weights range from [0,1], and additional algorithms are needed to implement the photonic matrix multiplication that includes negative numbers.

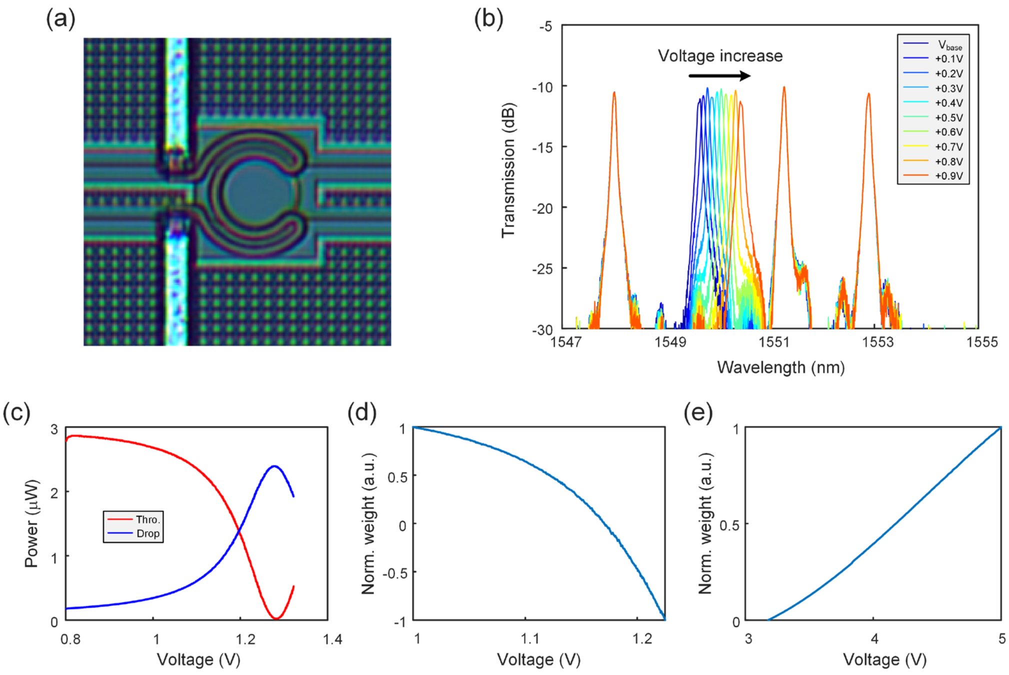

Fig. S2. Characterization of the microring and the modulator. **a** Microscope image of the microring. **b** Transmission spectrum of a four-microring synapse. With the base voltage applied, different voltages (0~1 V with 0.1 V/step) are additionally applied on the second microring. **c** Optical power of the through port and the drop port of the microring. **d** Normalized weight of the microring. **e** Normalized weight of the modulator.

S4. Control flow of the self-calibrating microring

Self-calibrating microring is a novel concept proposed in our recent work [1] to represent microring with the ability to automatically calibrate weights without human intervention. The specific control flow is shown in Fig. S3. In addition to the main wavelength (λ_1) that performs the computation task, an additional monitoring wavelength (λ_M) is introduced to monitor the weight of the microring in real time and calibrate the weight in time when error occurs. The monitoring wavelength is separated from the main wavelength by one FSR, and a mapping between the weights of the microring and the power of the monitoring wavelength can be established as a lookup table (W-P mapping). The advantage of W-P mapping is that it is not sensitive to temperature changes and overcomes the defect that the state of the microring is easily affected by the environment.

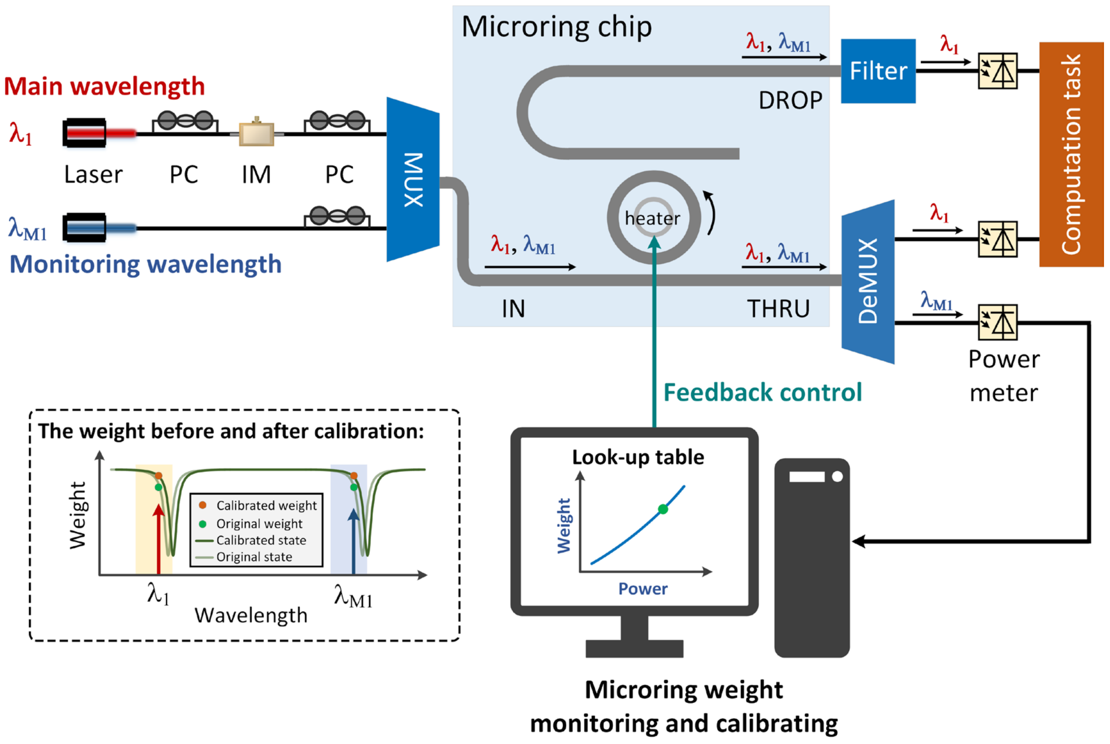

Fig. S3. The control flow of microring self-calibration.

S5. Design of multi-channel power supply

In recent years, the scale of photonic computing chips is increasing rapidly, which involves the amplitude and phase modulation of a large number of photonic devices, therefore it requires accurate control of multiple channels. A multi-channel power supply can be used to replace multiple individual power supplies, saving space and reducing the overall cost. To meet our experimental requirements, we developed a multi-channel power supply containing FPGA, DAC, and ADC. The FPGA is capable of interfacing with the ADC and DAC modules, and has enough logic resources to implement the required control functions. The DAC can generate the required output voltages (ranging from 0~10 V) for each channel, while the ADC is used to sample the output voltage of each channel with 16-bit resolution. In our FPGA-based control architecture, the number of DAC and ADC interfaces is extensible. The pluggable board equipped with DAC and ADC circuits can be connected to the main control FPGA through standard slots, and the number of channels can be extended according to actual needs.

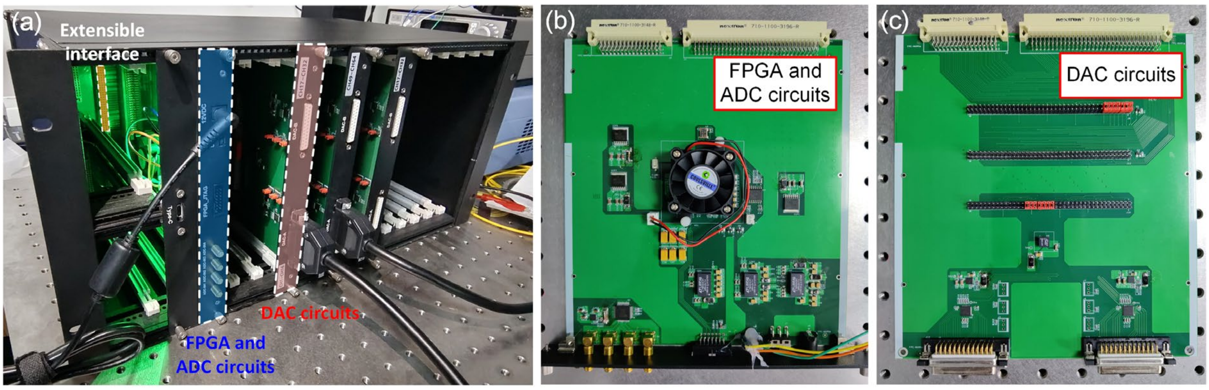

Fig. S4. Design of multi-channel power supply with FPGA, DAC, and ADC modules. **a** Prototype of the multi-channel power supply. **b** The pluggable board equipped with FPGA and ADC circuits. **c** The pluggable board equipped with DAC circuits.

For the scenario with the 16×16 weight bank, a custom electronic control module is required to drive the 16×16 weight bank and EO modulators. The packaged 16×16 photonic weight bank chip is shown in Fig. S5. In this protocol, high-speed electrical I/O is achieved through standard pluggable connecting finger interconnected with external control circuits instead of bulky Jumper wires.

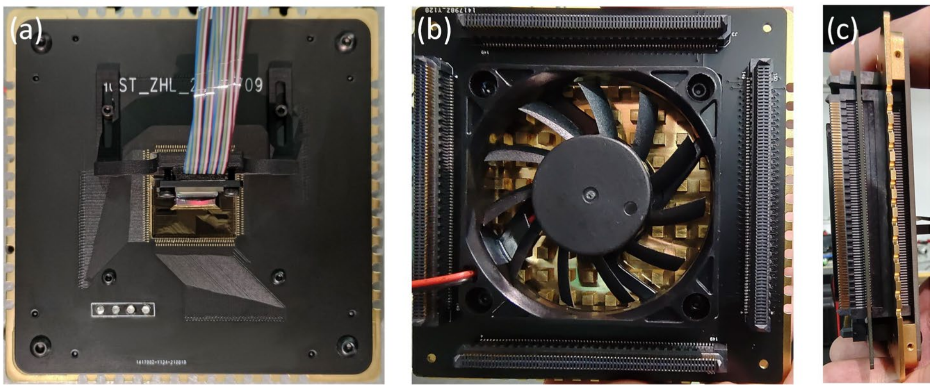

Fig. S5. Details of the front, back, and side of the packaged 16×16 photonic weight bank chip.

The FPGA model we use in this work is Kintex 7, which is a high-performance and low-power FPGA based on 28 nm high-k metal gate (HKMG) process technology developed by Xilinx. This FPGA model supports high-speed serial connections with built-in multi-gigabit transceivers from 600 Mb/s to maximum rate of 12.5 Gb/s, offering a special low-power mode, optimized for chip-to-chip interfaces. The number of I/O Pins is 500, which is suitable for demanding optical computing applications. In addition, this model supports DDR3 interfaces up to 1866 Mb/s and mainstream standards like PCIe and 10 Gigabit Ethernet. Detailed parameter information is shown in Table S1, and its datasheet can be obtained from Xilinx's official website: https://www.xilinx.com/products/silicon-devices/fpga/kintex-7.html#documentation.

Table S1 Technical information of the FPGA model.

**Max. capability**	**Kintex 7**
Logic cells	478 k
Block RAM	34 Mb
DSP slices	1920
DSP performance	2845 GMAC/s
Transceivers	32
Transceiver speed	12.5 Gb/s
Serial bandwidth	800 Gb/s
PCIe interface	x8 Gen2
Memory interface	1866 Mb/s
I/O pins	500
Package scheme	Bare-Die Flip-Chip and High-Performance Flip-Chip

S6. Technical information of the balanced photodetector

The balanced photodetector used in this work is BPDV2120R, which consists of two optimized 43 GHz waveguide-integrated photodiodes on a single chip. The hermetic module is especially designed for use in the optical window at 1550 nm and optimal RF performance. The pulse response reveals virtually no ringing. Detailed electro-optical specifications are shown in Table S2, and the datasheet can be found from the website: https://www.alldatasheet.com.

Table S2 Electro-optical specifications of the balanced photodetector.

Parameters	Symbol	Typical value
Photodiode DC responsivity	R	0.45 A/W
Imbalance of responsivity	Imb	0.15 dB
Polarization dependent loss	PDL	0.4 dB
Photodiode dark current	I_{dark}	5 nA
Optical return loss	ORL	27 dB
3dB cuf-off frequency	f_{3dB}	42 GHz
RF common mode rejection ratio	CMRR	18 dB
Output reflection coefficient	S22	-5 dB
Skew		2 ps
Skew (inter detector module)		10 ps
Noise equivalent power	NEP	$\sim 8.93\times10^{-13}\ W/\sqrt{Hz}$

S7. Photonic matrix multiplication including negative values

Optical computing architectures usually obtain the results of photonic matrix multiplication by detecting the optical power output from photonic chips [2-9]. However, since the power of light cannot be negative, it is necessary to design a matrix decomposition algorithm to realize the photonic matrix multiplication including negative numbers. For photonic matrix multiplication $O=XI$, where O is the

output matrix, X is the transmission matrix of the microring array, and I is the input matrix. The microring is designed as add-drop type, and negative numbers can be achieved by differential detection of the power at the through and drop ports. Therefore, only the input matrix I needs to be decomposed. First, the input matrix (real numbers) can be divided into I_+, including all the positive elements and zeros, and I_-, including all the absolute values of the negative elements. The relationship between I_+, and I_- can be expressed as

$$\begin{cases} I_+ = \dfrac{|I| + I}{2} \\ I_- = \dfrac{|I| - I}{2} \\ I = I_+ - I_- \end{cases} \qquad (S1)$$

These two non-negative matrices, I_+ and I_-, will be used to replace the origin input matrix I. The transmission matrix X is configured on the microring array and then multiplied with I_+ and I_-, respectively, to obtain two multiplication results XI_+ and XI_-. Finally, the results of photonic matrix multiplication containing negative numbers can be obtained by differencing them:

$$O = XI = X(I_+ - I_-) = XI_+ - XI_- \qquad (S2)$$

S8. Details of CNN training

Pytorch is a well-known neural network framework open sourced by Facebook (Meta), and is used to train our CNN model for human emotion recognition. The activation function used in our CNN model is ReLU, and the loss function is the cross-entropy. Fig. S6 shows the curves of loss function and accuracy with the number of training epochs.

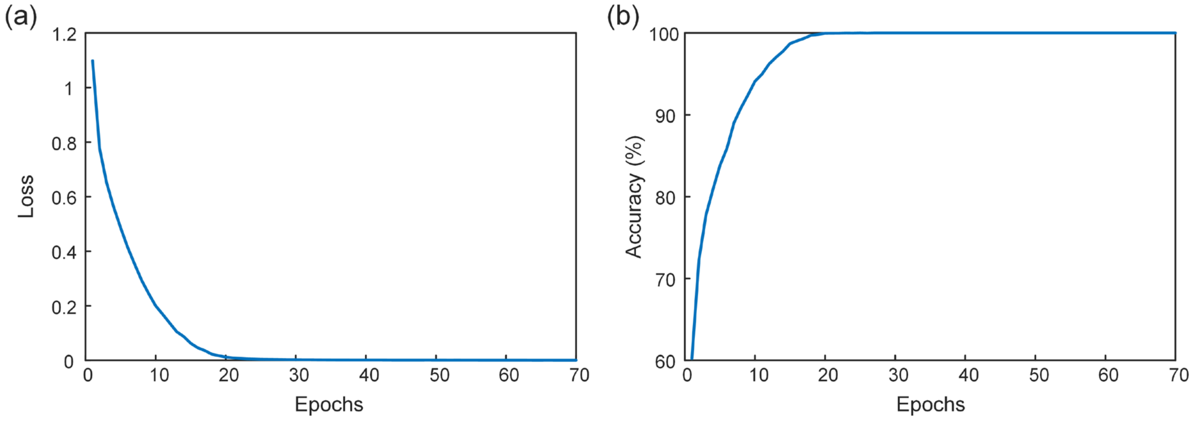

Fig. S6. Loss and accuracy during CNN training. **a** The loss curve. **b** The accuracy curve.

S9. Comparison of datasets used for photonic experimental validation

Photonic computing hardware has successfully verified some classical datasets, demonstrating its potential in performing deep learning tasks. Table S3 summarizes the datasets used for photonic experimental validation. To approximate real-world scenarios for deep learning, the dataset used in our work has much larger image size than others validated by photonic computing hardware, and facial expressions also have richer details than digits or letters. In addition, the dataset used in our work is open to the public and easily accessible to academic peers.

Table S3 Comparison of datasets used for photonic experimental validation.

Database	Image size	Image type	Accessibility	Photonic validation
MNIST [10]	28 × 28	Handwritten digits	To public	Ref. [5, 7, 11-13]
Fashion-MNIST [14]	28 × 28	Clothing	To public	Ref. [15, 16]
Three-class set	3 × 5	Pixelated letters	Only private	Ref. [17]
Four-class set	3 × 5	Pixelated letters	Only private	Ref. [18]
Two- or four-class sets	5 × 6	Handwritten letters	Only private	Ref. [19]
RAF-DB [20]	100 × 100	Facial expression	To public	Our work

S10. Impact of Gaussian noise on CNN inference accuracy

We test the impact of Gaussian noise on the CNN model and the results are presented in Fig. S7. The test is performed on a digital computer, Gaussian noise with different standard deviations (σ_{noise}) is added to the model, and the inference accuracy of the model on the test set is recorded. We can observe that when the Gaussian noise is small, the inference accuracy of the model is basically unchanged, which reflects the robustness of the neural network. However, once the standard deviation of the Gaussian noise is larger than 0.1, the inference accuracy will decrease significantly. Therefore, photonic computing hardware needs to achieve high accuracy to ensure that the error is within a tolerable range for the neural network model.

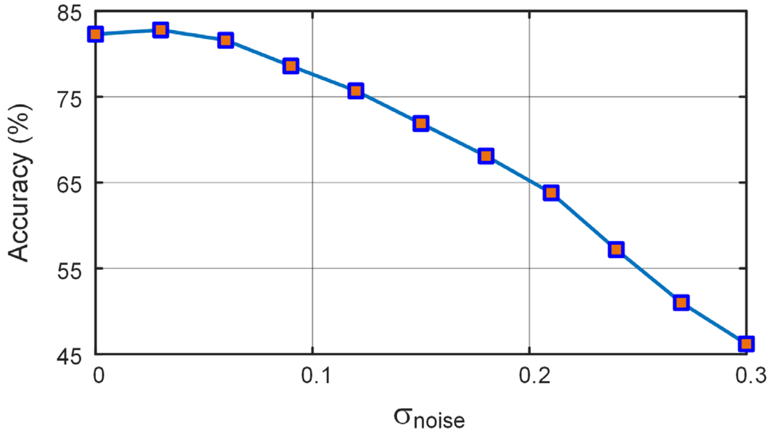

Fig. S7. Simulated accuracy of the CNN model with different standard deviations of additive Gaussian noise.

S11. Estimation of throughput, computing density, and energy consumption

Throughput, computing density, and energy consumption are key metrics to evaluate the performance of computing hardware. In the field of high performance computing (HPC), throughput is defined as the number of operations per second (OPS) when a processor computes, and is often used to describe the performance of a processor. The throughput of photonic computing hardware can be calculated by Eq. (S3)[2]:

$$T = 2m \times N^2 \times r\ OPS \qquad (S3)$$

where T is the throughput in units of OPS (the time spent during photonic computing does not include the time spent in off-chip signal loading), m is the number of layers implemented by photonic computing hardware, N^2 is the size of on-chip weight bank, and r is the detection rate of the PDs. Since the MIONN architecture naturally performs multiplication and accumulation (MAC) operations, and each MAC operation consists of one multiplication operation and one accumulation operation, one MAC operation corresponds to two operations. At a typical 100 GHz photonic detection rate, our MIONN proof-of-concept chip (N^2=4×4) and photonic engine (N^2=16×16) have throughputs of 3.2 TOPS and 51.2 TOPS, respectively. Based on the calculated throughput, the computing density of the MIONN architecture can be further calculated as 10.2 TOPS/mm^2 using Eq. (S4):

$$C = T/F \qquad (S4)$$

where C is the computing density, and F is the footprint of photonic computing core. The power consumption of the MIONN mainly includes integrated chips and external benchtop instruments. Integrated chips include on-chip optical comb generation, data modulation, on-chip weight bank, PDs, TEC, and digital backend. External benchtop instruments include arbitrary waveform generator, oscilloscope, and the computer to train the neural network. According to our investigation, in recent high-level papers[5, 7, 9], the power budget only considers the optical power, modulator, photonic computing core, and TEC module, while the power consumption of external benchtop instruments such as arbitrary waveform generator, oscilloscope, and computer to train the neural network is usually not included in the power consumption estimation. In order to make a relatively fair comparison with other works in the field of optical computing, the expected power budget for integrated chips is calculated using similar methods in ref.[5, 7, 9], and the overall power budget with external benchtop instruments is also provided in the Table S4. The total power consumption of the MIONN is estimated to be about 40.52 W (N=4) and 86.12 W (N=16), and detailed power information of the components in each module is given in Table S4. Regarding the whole system, the energy efficiency of the MIONN can be obtained as 78.97 GOPS/W (N=4) and 594.52 GOPS/W (N=16). We can observe that most power consumption comes from bench-top instruments, and the energy efficiency of MIONN can be significantly improved by increasing the scale of the on-chip weight bank. Considering only the optical comb and weight bank chips, the energy efficiency of the MIONN can be obtained as 1.21 TOPS/W (N=4) and 4.18 TOPS/W (N=16).

Table S4 Estimated power consumption of the MIONN.

Module	Components	Power (W)
On-chip comb generation	Pump laser	1
	Auxiliary laser	1
Data modulation	Modulator driver (N=4)	2.5×4=10
	Modulator driver (N=16)	2.5×16=40
On-chip microring weight bank	On-ring heaters (N^2=4×4)	0.04×16=0.64
	On-ring heaters (N^2=16×16)	0.04×256=10.24
Photodetectors	PD driver (N=4)	0.5×4=2
	PD driver (N=16)	0.5×16=8
TEC	TEC for pump laser	1.5
	TEC for microcomb	1.5
	TEC for on-chip weight bank	2.88
Digital backend	FPGA control circuits	20
External benchtop instruments	Arbitrary waveform generator	50
	Oscilloscope	120
	Computer to train the network	130
Total power consumption (include benchtop instruments)	N=4	340.52
	N=16	386.12
Total power consumption (exclude benchtop instruments)	N=4	40.52
	N=16	86.12

References

[1] J. Cheng, Z. He, Y. Guo, et al., "Self-calibrating microring synapse with dual-wavelength synchronization," *Photonics Res.*, vol. 11, no. 2, pp. 347-356, 2023.

[2] Y. Shen, N. C. Harris, S. Skirlo, et al., "Deep learning with coherent nanophotonic circuits," *Nat. Photonics*, vol. 11, no. 7, pp. 441-446, 2017.

[3] A. N. Tait, T. F. de Lima, E. Zhou, et al., "Neuromorphic photonic networks using silicon photonic weight banks," *Sci. Rep.*, vol. 7, no. 1, p. 7430, 2017.

[4] H. Zhou, Y. Zhao, G. Xu, et al., "Chip-Scale Optical Matrix Computation for PageRank Algorithm," *IEEE J. Sel. Top. Quantum Electron.*, vol. 26, no. 2, pp. 1-10, 2020.

[5] J. Feldmann, N. Youngblood, M. Karpov, et al., "Parallel convolutional processing using an integrated photonic tensor core," *Nature*, vol. 589, no. 7840, pp. 52-58, 2021.

[6] C. R. Huang, S. Fujisawa, T. F. de Lima, et al., "A silicon photonic-electronic neural network for fibre nonlinearity compensation," *Nat. Electron.*, vol. 4, no. 11, pp. 837-844, 2021.

[7] X. Xu, M. Tan, B. Corcoran, et al., "11 TOPS photonic convolutional accelerator for optical neural networks," *Nature*, vol. 589, no. 7840, pp. 44-51, 2021.

[8] S. Xu, J. Wang, S. Yi, and W. Zou, "High-order tensor flow processing using integrated photonic circuits," *Nat. Commun.*, vol. 13, no. 1, p. 7970, 2022.

[9] B. Bai, Q. Yang, H. Shu, et al., "Microcomb-based integrated photonic processing unit," *Nat. Commun.*, vol. 14, no. 1, p. 66, 2023.

[10] Y. LeCun, C. Cortes, and C. J. C. Burges. "The MNIST database of handwritten digits." http://yann.lecun.com/exdb/mnist/.

[11] H. Zhang, M. Gu, X. D. Jiang, et al., "An optical neural chip for implementing complex-valued neural network," *Nat. Commun.*, vol. 12, no. 1, p. 457, 2021.

[12] T. Wang, S.-Y. Ma, L. G. Wright, et al., "An optical neural network using less than 1 photon per multiplication," *Nat. Commun.*, vol. 13, no. 1, p. 123, 2022.

[13] T. Fu, Y. Zang, Y. Huang, et al., "Photonic machine learning with on-chip diffractive optics," *Nat. Commun.*, vol. 14, no. 1, p. 70, 2023.

[14] H. Xiao, K. Rasul, and R. Vollgraf, "Fashion-MNIST: a Novel Image Dataset for Benchmarking Machine Learning Algorithms," p. arXiv:1708.07747doi: 10.48550/arXiv.1708.07747.

[15] X. Lin, Y. Rivenson, N. T. Yardimci, et al., "All-optical machine learning using diffractive deep neural networks," *Science*, vol. 361, no. 6406, p. 1004, 2018.

[16] H. H. Zhu, J. Zou, H. Zhang, et al., "Space-efficient optical computing with an integrated chip diffractive neural network," *Nat. Commun.*, vol. 13, no. 1, p. 1044, 2022.

[17] Z. Wang, L. Chang, F. Wang, T. Li, and T. Gu, "Integrated photonic metasystem for image classifications at telecommunication wavelength," *Nat. Commun.*, vol. 13, no. 1, p. 2131, 2022.

[18] J. Feldmann, N. Youngblood, C. D. Wright, H. Bhaskaran, and W. H. P. Pernice, "All-optical spiking neurosynaptic networks with self-learning capabilities," *Nature*, vol. 569, no. 7755, pp. 208-214, 2019.

[19] F. Ashtiani, A. J. Geers, and F. Aflatouni, "An on-chip photonic deep neural network for image classification," *Nature*, vol. 606, no. 7914, pp. 501-506, 2022.

[20] S. Li, and W. Deng. "Real-world Affective Faces Database (RAF-DB)." http://www.whdeng.cn/RAF/model1.html.
